# Regular Strength and Sprint Training Counteracts Bone Aging: A 10‐Year Follow‐Up in Male Masters Athletes

**DOI:** 10.1002/jbm4.10513

**Published:** 2021-05-24

**Authors:** Tuuli H Suominen, Markku Alén, Timo Törmäkangas, Hans Degens, Jörn Rittweger, Ari Heinonen, Harri Suominen, Marko T Korhonen

**Affiliations:** ^1^ Gerontology Research Center, Faculty of Sport and Health Sciences University of Jyväskylä Jyväskylä Finland; ^2^ Department of Medical Rehabilitation, Oulu University Hospital and Center for Life Course Health Research University of Oulu Oulu Finland; ^3^ Department of Life Sciences, Musculoskeletal Science, and Sports Medicine Research Centre Manchester Metropolitan University Manchester UK; ^4^ Institute of Sport Science and Innovations Lithuanian Sports University Kaunas Lithuania; ^5^ Institute of Aerospace Medicine German Aerospace Center (DLR) Cologne Germany; ^6^ Department of Pediatrics and Adolescent Medicine University of Cologne Cologne Germany; ^7^ Faculty of Sport and Health Sciences University of Jyväskylä Jyväskylä Finland

**Keywords:** AGING, BONE pQCT, EXERCISE, HIGH‐IMPACT TRAINING, LONGITUDINAL STUDIES

## Abstract

Cross‐sectional and interventional studies suggest that high‐intensity strength and impact‐type training provide a powerful osteogenic stimulus even in old age. However, longitudinal evidence on the ability of high‐intensity training to attenuate age‐related bone deterioration is currently lacking. This follow‐up study assessed the role of continued strength and sprint training on bone aging in 40‐ to 85‐year‐old male sprinters (*n* = 69) with a long‐term training background. Peripheral quantitative computed tomography (pQCT)‐derived bone structural, strength, and densitometric parameters of the distal tibia and tibia midshaft were assessed at baseline and 10 years later. The groups of well‐trained (actively competing, sprint training including strength training ≥2 times/week; *n* = 36) and less‐trained (<2 times/week, no strength training, switched to endurance training; *n* = 33) athletes were formed according to self‐reports at follow‐up. Longitudinal changes in bone traits in the two groups were examined using linear mixed models. Over the 10‐year period, group‐by‐time interactions were found for distal tibia total bone mineral content (BMC), trabecular volumetric bone mineral density (vBMD), and compressive strength index, and for mid‐tibia cortical cross‐sectional area, medullary area, total BMC, and BMC at the anterior and posterior sites (polar mass distribution analysis) (*p* < 0.05). These interactions reflected maintained (distal tibia) or improved (mid‐tibia) bone properties in the well‐trained and decreased bone properties in the less‐trained athletes over the 10‐year period. Depending on the bone variable, the difference in change in favor of the well‐trained group ranged from 2% to 5%. The greatest differences were found in distal tibia trabecular vBMD and mid‐tibia posterior BMC, which remained significant (*p* < 0.05) after adjustment for multiple testing. In conclusion, our longitudinal findings indicate that continued strength and sprint training is associated with maintained or even improved tibial properties in middle‐aged and older male sprint athletes, suggesting that regular, intensive exercise counteracts bone aging. © 2021 The Authors. *JBMR Plus* published by Wiley Periodicals LLC on behalf of American Society for Bone and Mineral Research.

## Introduction

Although the ability of bone to adapt to physical exercise is most marked during youth, bone also retains some of its plasticity in later decades of life. However, participation in vigorous bone‐loading exercise typically decreases with aging,^(^ ^1^ ^)^ and reduced physical activity levels in old age likely contribute to the age‐related loss of bone mass. Middle‐aged and older masters athletes, although comprising only a small proportion of their cohort, provide a valuable model to study age‐related changes in bone in the presence of regular high‐intensity loading.^(^ ^2^ ^)^ According to previous investigations, sprint training combining running and supplementary jumping and strength exercises may provide the most powerful osteogenic training stimulus for the maintenance of bone mass and structural integrity with age, at least in the lower body skeleton.^(^ ^3^, ^4^, ^5^, ^6^, ^7^, ^8^, ^9^, ^10^, ^11^, ^12^ ^)^

The tibia has been the focus of many exercise studies. Using peripheral quantitative computed tomography (pQCT), we and others have observed that in middle‐aged and older masters sprint athletes, the indicators of bone strength of the distal and mid‐shaft regions of the tibia are above average, yet they nevertheless show an age‐related decline.^(^ ^8^, ^11^ ^)^ These cross‐sectional studies may not, however, accurately indicate the longitudinal effects of aging and training on bone. In a previous randomized controlled trial with masters sprinters, we found that by combining intensive strength exercises with sport‐specific sprint training, it is possible to improve mid‐tibia structure and strength by 2% to 3% even after a rather short training period (20 weeks).^(^ ^13^ ^)^ In addition, some studies have found significant changes in bone characteristics in response to high‐intensity strength and impact training in nonathletic older adults with low bone mass.^(^ ^14^ ^)^ Together, these studies suggest that the adaptability of bone to high‐intensity exercise is likely maintained during aging. The osteogenic adaptations in our previous study, as in other exercise trials in older people in general,^(^ ^14^, ^15^, ^16^, ^17^, ^18^, ^19^ ^)^ were modest. However, if intense strength and impact training is maintained on a regular basis from midlife to late adulthood, it could attenuate the aging‐related deterioration of bone structure and strength to ultimately reduce the risk of osteopenia and osteoporosis.

The present study expands our previous cross‐sectional and experimental findings by providing long‐term follow‐up data on the same study population. The purpose of the study was to examine 10‐year changes in pQCT‐derived bone structural, strength, and densitometric parameters of the distal tibia and tibial midshaft in 40‐ to 85‐year‐old male masters sprinters and, most importantly, to assess the role of continued sport‐specific sprint and strength training on the changes in bone traits. Owing to the wide age range of the participants, the results are also shown separately for the two age groups (40 to 64 and 65 to 85 years). An exploratory objective was to compare the changes in bone traits between cross‐sectional estimates and longitudinal analyses.

## Materials and Methods

### Design and participants

This 10‐year follow‐up study was part of a larger research program investigating the effects of age and long‐term sprint training on musculoskeletal characteristics and neuromuscular function among male masters athletes (ISRCTN17271498).^(^ ^11^, ^13^, ^20^ ^)^ The recruitment procedure and study design have been described in detail earlier.^(^ ^11^, ^13^ ^)^ Briefly, 83 male masters sprinters (aged 40 to 85 years) with a long‐term training background and success in international or national masters sprint events participated in the baseline measurements. To be eligible for the study, the athletes had to continue systematic training and competing in sprint events. Exclusion criteria included medications affecting bone metabolism.

Ten years later, the participants were recontacted by telephone and invited to participate in the follow‐up study. Sixty‐nine (83%) of the original 83 participants expressed willingness to continue in the study. Of the remaining participants, 6 had died, 3 could not be located, and 5 declined to participate because of poor health (*n* = 4) or lack of interest (*n* = 1). The main follow‐up measurements were carried out at the same time of year (November to December) as at baseline. However, 15 participants were unable to attend this study visit. Their pQCT data were later obtained as part of a bone examination carried out in the same laboratory during the World Masters Indoor Championships held in Jyväskylä in April of the same year. All participants provided a written informed consent before participation in the study. The study was approved by the ethical committees of the University of Jyväskylä and the Central Finland Health Care District and conformed with the principles of the Declaration of Helsinki.

Based on their training and competition status at the time of follow‐up, the athletes were categorized into two groups: well‐trained (*n* = 36) and less‐trained (*n* = 33). The well‐trained group comprised those who reported ongoing systematic strength and sprint training at least twice weekly during the preceding year, divided into indoor and outdoor seasons, and participation in international or national masters sprint events. The less‐trained group comprised those who reported strength and sprint training less than twice weekly, did no strength training, had retired from sport activities, had switched to endurance‐type training and competing in endurance events, or reported taking long‐term training breaks toward the end of the 10‐year follow‐up. Training frequency (main inclusion criterion for the well‐trained group) was assessed separately for different training modes (strength, sprint, and plyometric training) and had to include both strength and sprint/plyometric types of training. In addition, questions covering the whole 10‐year follow‐up period (timing and length of possible training breaks, possible changes in training habits, competition history) were utilized to confirm participants' training and competitive status. Training breaks were evaluated according to their assumed effect (length and proximity to follow‐up measurements) on the bone results. Based on our previous randomized controlled trial (RCT) with the same study population,^(^ ^13^ ^)^ where the exercise‐induced adaptations were likely derived from increased and intensified strength training, we were especially interested in the associations between strength training and bone aging. Hence, strength training was mandatory for an athlete to be categorized as well‐trained.

### Peripheral quantitative computed tomography

pQCT (XCT‐2000, Stratec Medizintechnik, Pforzheim, Germany) scans were obtained from the distal tibia and tibial midshaft of the dominant leg (the leg used for take‐off in a one‐footed jump) according to previously described methods.^(^ ^11^, ^21^ ^)^ The same scanner was used in all the baseline and follow‐up measurements. During the study, a daily quality assessment was performed using a standard phantom provided by the manufacturer. The distal tibia was scanned at 5% and the tibial midshaft at 50% of the tibia length proximal to the distal end plate. Tibia length was defined as the distance between the lateral malleolus and the lateral knee joint cleft. A single (2‐mm) axial slice with a pixel size of 0.8 × 0.8 mm, typical tube voltage of 46 kV, tube current of 0.3 mA, and scan speed of 20 mm/s was obtained. The cross‐sectional images were analyzed with the Geanie software program (version 2.1, Commit Ltd, Espoo, Finland). To determine the outer bone border, the segmentation threshold was set at 169 mg/cm^3^ for the distal tibia and at 280 mg/cm^3^ for the mid‐tibia. Separation of subcortical/trabecular and cortical bone was performed using an automatic contour detection algorithm (K‐mode). At the distal site, bone marrow was included in the analyses, whereas at the midshaft site, bone marrow was excluded by applying a threshold of 100 mg/cm^3^.

Total bone mineral content (BMC_TOT_, mg/mm), trabecular volumetric BMD (vBMD_TRAB_, mg/cm^3^), total cross‐sectional area (CSA_TOT_, mm^2^), and compressive bone strength index (BSI_COMP_, g^2^/cm^4^ = vBMD_TOT_^2^ × CSA_TOT_)^(^ ^22^ ^)^ were analyzed for the distal tibia. At the midshaft site, BMC_TOT_, cortical vBMD (vBMD_CO_), CSA_TOT_ (including bone marrow), cortical CSA (CSA_CO_), medullary CSA (CSA_M_ = CSA_TOT_‐CSA_CO_, including subcortical and medullary CSA), and density‐weighted moments of inertia (*I*_*max*_ and *I*_*min*_, mg*cm), reflecting the bone's resistance to bending in the direction of the greatest and smallest flexural rigidity, were determined. In addition, BMC was further analyzed as the polar distribution of bone mineral mass around its center, using 5° steps that were subsequently averaged into eight 45° sectors: anterior (A), anteromedial (A‐M), medial (M), posteromedial (P‐M), posterior (P), posterolateral (P‐L), lateral (L), and anterolateral (A‐L). The root mean square coefficient of variation (CV_RMS_) for BMD, structure and strength index measurements in our laboratory ranges from 0.4 to 1.6%.^(^ ^23^ ^)^

Muscle CSA (mm^2^) at the 50% site was analyzed by manually drawing along the outer boundary of the calf and applying thresholds of 11 and 280 mg/cm^3^ to exclude fat and bone.

### Anthropometry, health, training, and sprint performance

At baseline and at follow‐up, the same methods were used to collect anthropometric, health, training, and sprint performance characteristics. Body height and mass were measured using a standard height gauge and a digital scale. Lean body mass (LBM) was assessed with a bioimpedance device (Spectrum II, RJL Systems, Detroit, MI, USA). Training status, health history, and current health of the athletes were assessed with a questionnaire and confirmed in a short interview and clinical examination. The questionnaire included detailed questions about current (during the preceding year, divided into indoor and outdoor seasons) and former training, competition performance, and injuries or diseases hindering physical training. At follow‐up, the questionnaire also included items on long‐term training breaks or significant decreases in the volume of strength and sprint training during the 10‐year follow‐up period. This data were utilized in the group allocation and are not reported in detail in this article. The health questionnaire included items on chronic diseases, medical operations, use of medical drugs and hormones, and smoking history. A 60‐m sprint time on an indoor synthetic track with spiked shoes was obtained using double‐beam photocell gates (starting line 0.7 m behind the first photocell gates). Own standing start without commands was used.

### Statistical analysis

Data are presented as mean values and standard deviations (SD) or 95% confidence intervals (CI) and additionally with CIs with alpha‐level adjustment for 19 simultaneous tests for the main analysis. Baseline physical and training characteristics of the well‐trained and less‐trained athletes were compared by independent samples *t* test. The association of continued strength and sprint training with longitudinal changes in bone outcomes was assessed based on an interaction term (group × time) in linear mixed models adjusted for age. The longitudinal changes in physical and training characteristics in the two groups were also examined using a similar approach. Neither the original randomization group nor anthropometric data were included in the bone outcome analyses because these were not associated with training status or the longitudinal changes in bone. One athlete was removed from the mid‐tibia analysis owing to movement artifact. Figures show individual and mean changes in the bone variables standardized with respect to their baseline measurement. Owing to the wide age range of the participants and possible differences in their training habits and/or responses, these changes were also calculated, as a sensitivity analysis, separately for two age groups aged 40 to 64 years (well‐trained, *n* = 21; less‐trained, *n* = 18) and 65 to 85 years (*n* = 15 and 15, respectively). The division into these age groups is based on our previous RCT^(^ ^13^ ^)^ with the same cohort. Finally, as an additional supplementary illustration, we compared the changes at follow‐up with the changes predicted from the cross‐sectional data. The point estimates and 95% CIs of the longitudinal 10‐year changes within individuals in bone traits were compared with the 10‐year predicted changes in estimated marginal means (% per decade) computed from cross‐sectional linear models with baseline bone traits as the dependent variable and continuous age as the predictor. Descriptive analyses were performed using SPSS 24.0 software (IBM Corp., Armonk, NY, USA) and the parameters of the linear mixed models were estimated and model‐derived statistics computed with custom scripts utilizing the nlme (version 3.1‐148) and emmeans packages (version 1.5.1) in R version 3.5.1 (R core team, Vienna, Austria).

The significance level was set at 5%. For the descriptive analysis, we report nominal *p* values and for the mixed analyses, both nominal and multiplicity‐adjusted *p* values and 95% CIs. Conducting several tests on the same data set increases the risk of false positives, whereas the conservative methods used to correct for multiple correlated tests tend to reject true positives along with false ones. For this reason, we utilized a correction procedure introduced by Cheverud^(^ ^24^ ^)^ that replaces the observed number of independent tests with their effective number. The effective number of tests is based on the independent number of sources of variability approximated by the eigenvalues of the outcome correlation matrix. Because the main tests for our analysis focus on changes over time (interactions), we used the correlation matrix of the follow‐up differences (follow‐up baseline) in computing the number of effective comparisons, *M*_*eff*_. We adopted the convention introduced by Nyholt^(^ ^25^ ^)^ and call *M*_*eff*_ the number of effective comparisons and the significance level 1 – (1 – α)^1/*Meff*^ the *M*_*eff*_‐Šidák‐corrected significance level. The approximate number of tests for the 19 outcomes was 16, yielding a *M*_*eff*_‐Šidák‐adjusted alpha of 0.00317. Standard errors for CIs for mean changes were computed based on the multiparameter version of the delta method (see, eg, Raykov and colleagues^(^ ^26^ ^)^).

## Results

### Physical and training characteristics

The baseline and 10‐year follow‐up characteristics of the athletes are shown in Table 1. {TBL 1} Mean follow‐up time was 9.8 ± 0.2 years. No differences between the groups of well‐trained and less‐trained athletes were observed in baseline physical and training characteristics except in the frequency of strength training, which was significantly higher in the well‐trained group (*p* = 0.002). Equally, no between‐group differences over time were observed in these outcomes. Mean training years at baseline were 31.5 (SD 16.0) for the well‐trained and 30.9 (16.4) for the less‐trained (*p* = 0.897) athletes. At 10 years, only a subsample of the participants completed the sprint performance and LBM measurements, as these assessments were conducted only during the main follow‐up measurements, which 15 participants were unable to attend. In addition, 13 participants did not participate in the sprint performance testing because of a musculoskeletal disorder (*n* = 8) or a chronic medical condition (*n* = 5).

**Table 1:** Baseline and Follow‐Up Physical, Training, and Bone Characteristics of Well‐Trained and Less‐Trained Athletes

	Baseline		10 years	
	Well‐trained (*n* = 36)	Less‐trained (*n* = 33)	Well‐trained (*n* = 36)	Less‐trained (*n* = 33)
Age (years)	60.8 (9.5)	60.5 (12.7)	70.6 (9.4)	70.4 (12.7)
Height (cm)	174 (6)	176 (6)	173 (6)	175 (7)
Mass (kg)	73.6 (7.0)	73.4 (7.8)	73.2 (7.9)	74.5 (8.8)
Lean body mass (kg)	63.2 (6.4)	62.9 (5.8)	62.1 (5.9)^a^	61.5 (6.5)^b^
Muscle CSA (mm^2^)	6763 (852)	6858 (1129)	6764 (923)	6893 (1265)
60‐m sprint time (s)	8.36 (0.58)^c^	8.63 (0.94)	9.32 (1.09)^b^	9.94 (2.45)^d^
Training frequency (sessions/wk)	4.5 (1.2)	4.3 (1.3)	4.2 (1.3)	3.3 (1.5)
Running and plyometrics (times/wk)	3.4 (1.5)	2.9 (1.6)^e^	2.1 (0.6)	0.8 (1.3)
Strength training (times/wk)	1.1 (0.7)	0.6 (0.6)^e^	1.4 (0.7)	0.7 (1.1)
Tibia 5%				
BMC_TOT_ (mg/mm)	427 (64)	420 (70)	425 (65)	405 (73)
CSA_TOT_ (mm^2^)	1195 (139)	1215 (172)	1192 (132)	1208 (175)
vBMD_TRAB_ (mg/cm^3^)	315 (39)	300 (38)	314 (41)	291 (43)
BSI_COMP_ (g^2^/cm^4^)	1.55 (0.39)	1.48 (0.39)	1.54 (0.41)	1.39 (0.40)
Tibia 50%				
CSA_TOT_ (mm^2^)	592 (60)	599 (71)	598 (59)	605 (73)^e^
CSA_CO_ (mm^2^)	416 (50)	416 (46)	420 (48)	410 (51)^e^
CSA_M_ (mm^2^)	177 (43)	183 (44)	178 (47)	195 (45)^e^
*I*_*max*_ (mg*cm)	4920 (1004)	5013 (1281)	5080 (1012)	5072 (1291)^e^
*I*_*min*_ (mg*cm)	1783 (384)	1849 (430)	1788 (367)	1862 (459)^e^
BMC_TOT_ (mg/mm)	508 (58)	511 (58)	513 (58)	506 (61)^e^
vBMD_CO_ (mg/cm^3^)	1095 (24)	1096 (26)	1093 (34)	1097 (30)^e^

Muscle CSA = muscle cross‐sectional area; BMC_TOT_ = total bone mineral content; CSA_TOT_ = total CSA; vBMD_TRAB_ = trabecular volumetric bone mineral density; BSI_COMP_ = compressive bone strength index; CSA_CO_ = cortical CSA; CSA_M_ = medullary CSA; *I*_*max*_, *I*_*min*_ = density‐weighted maximal and minimal moments of inertia; vBMD_CO_ = cortical vBMD.

Values are means (SD). Note: 15 participants were unable to attend the main follow‐up measurements when lean body mass and sprint performance were assessed.

^a^ *n* = 31.

^b^ *n* = 25.

^c^ *n* = 34.

^d^ *n* = 16.

^e^ *n* = 32.

None of the participants reported taking any medications that affected bone metabolism. Three participants in the well‐trained and 3 in the less‐trained group presented with prostate cancer. All participants were free of other diseases that could affect bone, such as rheumatoid arthritis, celiac disease, or colitis ulcerosa. One current smoker was found in the well‐trained group and 6 former smokers in each group (12 former smokers in total).

### Bone traits

No differences were observed in baseline bone characteristics between the well‐trained and less‐trained (Table 1) except for distal tibia BMC_TOT_ and vBMD_TRAB_, which were significantly higher in the 40‐ to 64‐year‐old well‐trained than less‐trained group (Supplemental Table S1).

**Fig. 1:**
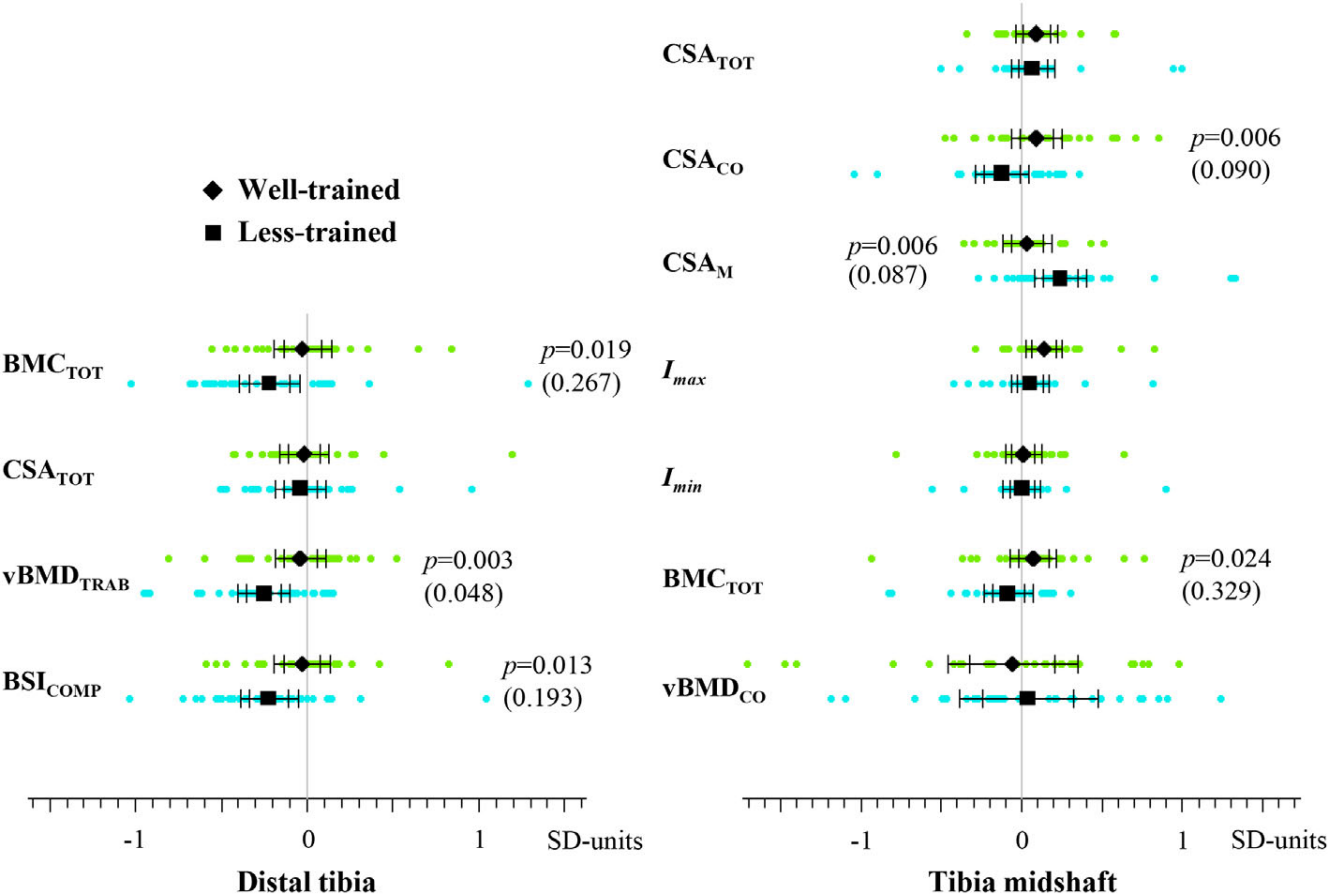
Ten‐year changes in distal tibia (*A*) and tibia midshaft (*B*) in well‐trained and less‐trained athletes. Outcomes were standardized with respect to their baseline values. Individual data points, group means, and 95% confidence intervals (CIs) for unadjusted (narrower CIs) and M_eff_‐Sidák multiple test‐corrected (wider CIs) analyses are presented. The displayed *p* values denote the unadjusted group × time interaction effect if *p* < 0.05. Multiplicity adjusted *p* values are shown in parentheses. Cases in the well‐trained group with vBMD_CO_ = −3.36 and 2.54 were cropped from the figure on the right‐hand side.

The associations of continued strength and sprint training with changes in the distal tibia bone traits are shown in Table 2, {TBL 2} Fig. 1, {FIG1} and Supplemental Fig. S1. At the distal tibia site, a significant group × time interaction was found for vBMD_TRAB_ (*p* = 0.003, raw value). vBMD_TRAB_ was maintained in the well‐trained and decreased (−3.2%) in the less‐trained athletes over the 10‐year period (Fig. 1). At follow‐up, the mean difference in the change in vBMD_TRAB_ in favor of the well‐trained was 2.8% (Fig. 1). A similar pattern was found for BMC_TOT_ and BSI_COMP._ In the well‐trained group, BMC_TOT_ and BSI_COMP_ were maintained, whereas in the less‐trained group they decreased by 3.5% and 5.9%, respectively (Fig. 1). The corresponding differences in change in favor of the well‐trained were 3.1% and 5.2%. After adjustment for multiple testing, the difference in vBMD_TRAB_ between the groups remained significant.

**Table 2:** Associations of Continued Strength and Sprint Training With Changes in Distal Tibia Bone Traits of the Masters Athletes

	Group	BL	10‐year change	Multiple testing					
				Unadjusted			Adjusted		
				95% CI		Group × time	95% CI		Group × time
BMC_TOT_ (mg/mm)	WT	427	−1.7	−9.1	5.7	0.019	−13.8	10.5	0.267
	LT	420	−14.5	−22.3	−6.8		−27.3	−1.8	
CSA_TOT_ (mm^2^)	WT	1195	−2.8	−17.1	11.5	0.743	−26.3	20.8	1.000
	LT	1209	−6.2	−21.1	8.8		−30.8	18.4	
vBMD_TRAB_ (mg/cm^3^)	WT	315	−1.5	−5.2	2.2	0.003	−7.5	4.6	0.048
	LT	300	−9.7	−13.6	−5.9		−16.0	−3.4	
BSI_COMP_ (g^2^/cm^4^)	WT	1.55	−0.01	−0.05	0.03	0.013	−0.08	0.06	0.193
	LT	1.47	−0.09	−0.13	−0.04		−0.16	−0.02	

BL = baseline; CI = confidence interval; WT = well‐trained (*n* = 36); LT = less‐trained (*n* = 33); BMC_TOT_ = total bone mineral content; CSA_TOT_ = total cross‐sectional area; vBMD_TRAB_ = trabecular volumetric bone mineral density; BSI_COMP_ = compressive bone strength index.

Values are estimated means. 95% CI for absolute change.

**Fig. 2:**
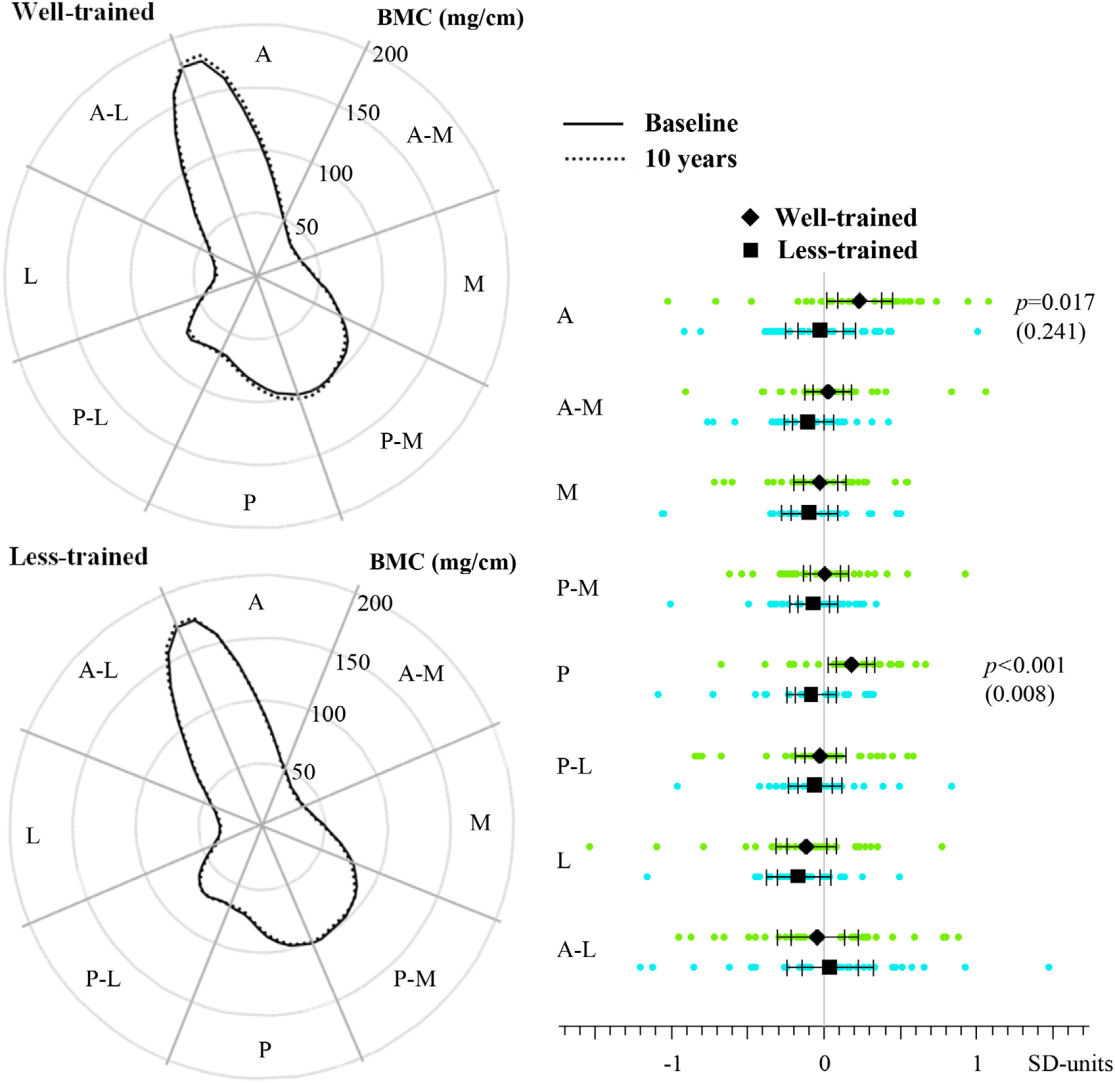
(*A*) Mean polar mass distribution curves for the well‐trained (upper panel) and less‐trained (lower panel) athletes at baseline and at the 10‐year follow‐up indicating the angular distribution of bone mineral mass around the center of mass in 5° steps that were subsequently averaged into eight 45° sectors. A = anterior; A‐M = anteromedial; M = medial; P‐M = posteromedial; P = posterior; P‐L = posterolateral; L = lateral; A‐L = anterolateral. (B) Ten‐year changes in polar mass distribution of the tibial shaft in well‐trained and less‐trained. Outcomes were standardized with respect to their baseline values. Individual data points, group means, and 95% confidence intervals (CIs) for unadjusted (narrower CIs) and M_eff_‐Sidák multiple test‐corrected (wider CIs) analyses are presented. The displayed *p* values denote the unadjusted group × time interaction effect if *p* < 0.05. Multiplicity adjusted *p* values shown in parentheses.

The associations of continued strength and sprint training with changes in the tibial mid‐shaft bone traits are shown in Tables 3 and 4, {TBL 3}{TBL 4} Figs. 1 and 2, {FIG2} and Supplemental Figs. S1 and S2. A significant group × time interaction was found for CSA_CO_ (*p* = 0.006, raw value) and BMC_TOT_ (*p* = 0.019, raw value). This reflected the increase in CSA_CO_ (+1.1%) and BMC_TOT_ (+0.8%) in the well‐trained and the decrease in both parameters (−1.4% and −0.9%, respectively) in the less‐trained athletes over the 10‐year period (Fig. 1). A significant group × time interaction found for CSA_M_ (*p* = 0.006, raw value) was reflected in the maintained CSA_M_ in the well‐trained and increased CSA_M_ (+4.9%) in the less‐trained athletes over the follow‐up. The mean difference in change in favor of the well‐trained was 2.5% for CSA_CO_, 1.8% for BMC_TOT_, and 4.2% for CSA_M_ (Fig. 1). After adjustment for multiple testing, the interactions were no longer significant, although CSA_CO_ and CSA_M_ showed an increasing trend in the well‐trained compared with less‐trained athletes (*p* = 0.090 and *p* = 0.087, respectively).

**Table 3:** Associations of Continued Strength and Sprint Training With Changes in Tibial Mid‐Shaft Bone Traits of the Masters Athletes

	Group	BL	10‐year change	Multiple testing					
				Unadjusted			Adjusted		
				95% CI		Group × time	95% CI		Group × time
CSA_TOT_ (mm^2^)	WT	518	6.0	0.6	11.5	0.724	−3.0	15.0	1.000
	LT	524	4.6	−1.2	10.4		−4.9	14.1	
CSA_CO_ (mm^2^)	WT	416	4.5	−0.4	9.5	0.006	−3.7	12.7	0.090
	LT	416	−5.8	−11.1	−0.6		−14.5	2.9	
CSA_M_ (mm^2^)	WT	205	1.5	−2.8	5.8	0.006	−5.6	8.5	0.087
	LT	212	10.4	5.9	15.0		3.0	17.9	
*I*_*max*_ (mg*cm)	WT	4918	161	76	245	0.109	22	299	0.845
	LT	5014	61	−28	150		−86	208	
*I*_*min*_ (mg*cm)	WT	1782	5.0	−24	34	0.852	−43	53	1.000
	LT	1861	1.0	−30	32		−50	52	
BMC_TOT_ (mg/mm)	WT	508	4.3	−1.1	9.6	0.024	−4.6	13.1	0.329
	LT	511	−4.8	−10.5	0.9		−14.2	4.6	
vBMD_CO_ (mg/cm^3^)	WT	1095	−1.4	−8.0	5.2	0.617	−12.2	9.5	1.000
	LT	1096	1.0	−6.0	8.0		−10.5	12.5	

BL = baseline; CI = confidence interval; WT = well‐trained (*n* = 36); LT = less‐trained (*n* = 32); CSA_TOT_ = total cross‐sectional area; CSA_CO_ = cortical CSA; CSA_M_ = medullary CSA; *I*_*max*_, *I*_*min*_ = density‐weighted maximal and minimal and moments of inertia; BMC_TOT_ = total bone mineral content; vBMD_CO_ = cortical volumetric bone mineral density.

Values are estimated means. 95% CI for absolute change.

**Table 4:** Associations of Continued Strength and Sprint Training With Changes in Polar Mass Distribution of the Tibial Shaft of the Masters Athletes

BMC	Group	BL	10‐year change	Multiple testing					
				Unadjusted			Adjusted		
				95% CI		Group × time	95% CI		Group × time
A	WT	913	29.4	11.5	47.3	0.017	−0.03	58.8	0.241
	LT	894	−2.6	−21.6	16.4		−33.8	28.6	
A‐M	WT	344	1.9	−4.4	8.1	0.077	−8.4	12.1	0.726
	LT	349	−6.4	−13.0	0.3		−17.3	4.6	
M	WT	476	−2.3	−12.1	7.5	0.419	−18.4	13.8	1.000
	LT	495	−8.1	−18.5	2.3		−25.2	9.0	
P‐M	WT	859	1.6	−12.8	15.9	0.283	−22.0	25.2	0.995
	LT	856	−9.8	−25.0	5.4		−34.8	15.3	
P	WT	727	25.8	11.9	39.7	<0.001	2.9	48.6	0.008
	LT	740	−11.4	−26.1	3.3		−35.6	15.3	
P‐L	WT	563	−2.1	−12.2	7.9	0.678	−18.6	14.4	1.000
	LT	554	−5.2	−15.9	5.4		−22.7	12.3	
L	WT	321	−6.5	−13.9	0.8	0.590	−18.6	5.6	1.000
	LT	327	−9.4	−17.2	−1.6		−22.2	3.4	
A‐L	WT	877	−5.2	−26.9	16.4	0.518	−40.9	30.4	1.000
	LT	892	5.0	−17.9	28.0		−32.7	42.8	

BMC = bone mineral content; BL = baseline; CI = confidence interval; WT = well‐trained (*n* = 36); LT = less‐trained (*n* = 32); A = anterior; A‐M = anteromedial; M = medial; P‐M = posteromedial; P = posterior; P‐L = posterolateral; L = lateral; A‐L = anterolateral.

Values are estimated means. 95% CI for absolute change. BMC – values (mg/cm) are sum values of nine 5° sectors.

The polar mass distribution of the tibial shaft showed a significant group × time interaction at the anterior and the posterior sites (Table 4 and Fig. 2). This was reflected in a site‐specific increase in BMC in the well‐trained and no change in the less‐trained athletes at follow‐up. In the well‐trained compared with less‐trained athletes, BMC_A_ increased by 3.5% and BMC_P_ by 5.1% (Fig. 2). After adjustment for multiple testing, BMC_P_ remained significant.

The mean changes in bone traits of the well‐trained and less‐trained athletes across the age groups are shown in Supplemental Figs. S1 and S2. The significant interactions (group × time, *p* < 0.05, raw values) and differences in changes in the bone outcomes observed in the main analyses (Figs. 1 and 2) were manifested in the age groups as follows. In the group aged 40 to 64 years, the mean difference in change in distal tibia bone traits in the well‐trained compared with less‐trained athletes was 3.2% for BMC_TOT_, 3.5% for vBMD_TRAB_, and 5.9% for BSI_COMP_. These manifested as maintained bone properties in the well‐trained and decreased bone properties in the less‐trained athletes over the 10‐year follow‐up period (Supplemental Fig. S1). In the group aged 65 to 85 years, the 4.1% difference in change in mid‐tibia CSA_CO_ was reflected as maintained CSA_CO_ in the well‐trained and decreased CSA_CO_ in the less‐trained athletes, whereas the 5.3% difference in change in CSA_M_ comprised no change in the well‐trained and an increase in the less‐trained athletes (Supplemental Fig. S1). Among the 40‐ to 64‐year‐olds, BMC_A_ and BMC_P_ increased over time in the well‐trained group and were maintained in the less‐trained group (Supplemental Fig. S2). The difference in change in favor of the well‐trained was 5.2% in BMC_A_ and 3.9% in BMC_P_. Among the 65‐ to 85‐year‐olds, the difference in change in BMC_P_ at follow‐up was 6.2%, comprising an increase in the well‐trained and a decrease in less‐trained athletes over the follow‐up (Supplemental Fig. S2).

In general, the longitudinal changes in bone traits did not follow the cross‐sectional trends predicted by the athletes’ baseline values (estimated cross‐sectional changes in comparison to longitudinal changes; Supplemental Table S2). For the distal tibia site, the mean change per decade predicted by the cross‐sectional analysis was −3.4% (range −7.2% [BSI_COMP_] to 0.5% [CSA_TOT_]) for all participants compared with the −1.8% (range −3.1% [BSI_COMP_] to −0.4% [CSA_TOT_]) found by the longitudinal analysis. For the midshaft site, the corresponding changes were −3.4% (range −6.5% [*I*_*max*_] to −0.3% [vBMD_CO_]) and 0.5% (range −0.1% [CSA_CO_] to 2.3% [*I*_*max*_]).

## Discussion

In this 10‐year follow‐up study of middle‐aged and older male sprint athletes, we found that regularly continued strength and sprint training was associated with maintained distal tibia trabecular density and with improved tibial midshaft bone mass at the posterior site (polar mass distribution analysis). In addition, a trend was found for an increased mid‐tibial cortical area in the well‐trained compared with less‐trained athletes, whereas the medullary area was maintained in the well‐trained and increased in the less‐trained athletes over the follow‐up. In the unadjusted analyses, significant group differences were found in distal tibia trabecular density, bone mass, and compressive strength, and in mid‐tibial cortical area, medullary area, bone mass, and BMC in the anteroposterior direction.

Longitudinal analysis of bone traits in masters sprint/power athletes has been limited to a single investigation^(^ ^27^ ^)^ and no previous data are available on the importance of sustained sport‐specific training on bone changes with aging. In line with our present findings, a recent 4‐year longitudinal study by Ireland and colleagues^(^ ^27^ ^)^ found greater maintenance of distal (4%) and mid‐tibial (66%) BMC in masters power (sprinting and jumping) than endurance athletes aged 37 to 85 years. At the distal site, the differences resulted from better maintenance of trabecular BMD, whereas at mid‐tibia, they were explained by the maintenance of cortical thickness and cortical BMD.^(^ ^27^ ^)^ Longitudinal studies conducted on middle‐aged and older masters long‐distance runners have shown maintained areal BMD at the hip and spine^(^ ^28^, ^29^ ^)^ but have not examined changes in bone structure, strength, and volumetric density.

In the present study, as in previous cross‐sectional^(^ ^7^, ^9^, ^11^ ^)^ and experimental studies^(^ ^13^ ^)^ on masters athletes and a twin study,^(^ ^21^ ^)^ the adaptations in cortical bone at the mid‐tibia site were mostly structural, whereas in the distal tibia, maintained bone strength was related to densitometric adaptations in trabecular bone. In our previous RCT with the same study population,^(^ ^13^ ^)^ we did not find exercise‐induced adaptations in the distal tibia, which was not surprising given the brevity of the intervention in these highly trained participants. In the present 10‐year follow‐up study, in accordance with recent findings on the positive effects of high‐intensity strength and impact training on nonathletic middle‐aged and older men with low bone mass,^(^ ^14^ ^)^ distal tibia BMC_TOT_, trabecular vBMD, and BSI_COMP_ were maintained in the well‐trained and decreased in the less‐trained athletes. The association with trabecular density remained significant even after adjusting for multiple testing. Overall, the more pronounced densitometric changes found in the 40‐ to 64‐year‐old group of athletes could be explained by the higher vertical compression forces exerted during impact‐type training. It is well known that normal aging processes impose limitations on training tolerance (eg, reduced recovery) and that many masters competitors are unable to maintain their absolute training intensity and volume as they enter old age.^(^ ^30^, ^31^, ^32^, ^33^, ^34^ ^)^ Even in the well‐trained group, absolute training intensities were likely lower in the older than younger athletes, although the relative training load might have been similar.

The adaptations at the mid‐tibia site were manifested as increased direction‐specific bone mass, which reflects the site‐specific nature of the observed increase in BMC_TOT_ and cortical area. In the well‐trained athletes, bone mass and cortical CSA increased, while in the less‐trained they declined. These improvements in bone mass and structure without increases in vBMD are in line with our previous RCT on masters athletes^(^ ^13^ ^)^ and with other exercise trials on aging nonathletes.^(^ ^35^, ^36^, ^37^ ^)^ The number of trials focusing on bone structure and strength among aging people is, however, limited, and studies have reported conflicting results,^(^ ^38^, ^39^ ^)^ possibly owing to short training periods and/or less‐intensive training regimens. In the present long‐term follow‐up, our sample included athletes who were able and competitively motivated to train at high intensities, enabling us to examine the long‐term association of training on bone aging. In contrast to the changes at the distal site, we observed more pronounced structural improvements at the mid‐tibia site in the 65‐ to 85‐year‐olds, indicating that the bending (and torsional) loading derived from strength and plyometric training may be an effective way to preserve bone even in old age. Moreover, the beneficial effect of such training on muscles (muscle mass, strength, and power) may further improve bone not only through increased loading from muscle contraction but possibly also through diverse (mechanical and non‐mechanical) muscle‐bone interactions.^(^ ^40^, ^41^ ^)^ In line with longitudinal findings on masters power and endurance athletes,^(^ ^27^ ^)^ muscle CSA measured at the mid‐tibia site did not correlate with training status or changes in bone. We suggest that the calf muscles may not adequately reflect the differences in the effects of training on the tibia, which is more likely affected by muscle pull from the knee extensors than by the muscles located at the tibial site.^(^ ^42^ ^)^ The knee flexors, which are highly important muscles in sprint performance, may also affect the tibia.^(^ ^43^ ^)^

According to the bone mass distribution of the midshaft, the well‐trained group showed increased bone mass in the A‐P direction, as also found in previous cross‐sectional athlete^(^ ^11^, ^44^, ^45^ ^)^ and twin studies^(^ ^21^ ^)^ and in an RCT combining hormone‐replacement therapy with high‐impact training.^(^ ^46^ ^)^ In those studies, the site‐specific increase in bone mass was seen as an increase in direction‐specific bending strength at the maximum axis (*I*_*max*_). The increase in *I*_*max*_ probably relates to posterior bending, which is the habitual loading pattern during sprint training and other weight‐bearing activities.^(^ ^47^ ^)^ In the present study, in accordance with recent longitudinal findings on masters athletes by Ireland and colleagues,^(^ ^27^ ^)^ *I*_*max*_ increased in both groups, although the well‐trained athletes showed a trend to a greater increase. The overall increase in *I*_*max*_ and *I*_*min*_ may also reflect age‐related endocortical resorption and compensatory periosteal apposition, ie, shift of the cortex further from the neutral axis.^(^ ^48^ ^)^ This is also supported by the overall increase in total bone area observed in the present study. The increases in *I*_*max*_ and CSA_TOT_ were less evident in the older less‐trained group of athletes, which further supports the benefits of regular training in old age.

The longitudinal changes in bone traits in the present study were relatively small. Because we did not include sedentary controls, direct comparisons with non‐exercisers cannot be made. However, previous longitudinal studies on non‐exercising older men are available.^(^ ^49^, ^50^ ^)^ Although not fully comparable with our results because of the different imaging method (high‐resolution [HR]‐pQCT) used, Burt and colleagues^(^ ^49^ ^)^ reported an annual decline of 0.3% to 1.1% in distal tibia density in men older than age 50 years. Similarly, by combining cross‐sectional and longitudinal data, Lauretani and colleagues^(^ ^50^ ^)^ observed significant lifetime decreases (approximately −20% between ages 20 and 100 years) in distal tibia total and trabecular vBMD measured by pQCT. In the present study, the mean decrease per decade in distal tibia vBMD_TRAB_ was 0.5% in the well‐trained and 3.5% in the less‐trained athletes.

At the mid‐tibia site, Lauretani and colleagues^(^ ^50^ ^)^ reported slight age‐related increases in cortical and total CSA, particularly before midlife. However, estimated bending strength declined over the life span. Continuous periosteal apposition was reported, especially during young adulthood and midlife. In the present study, no significant increase in total CSA in the well‐trained compared with less‐trained athletes was found, whereas medullary area increased in the less‐trained but remained unchanged in the well‐trained athletes. Together, these observations suggest that exercise‐induced adaptations were more likely to occur in the endocortical than periosteal surfaces, reflecting reduced endocortical bone loss in the well‐trained athletes. This accords with an animal study by Birkhold and colleagues^(^ ^51^ ^)^ suggesting that the mechanoresponsiveness of the endocortical surface is better preserved during aging than the periosteal surface. Overall, the age‐related changes in the above‐mentioned longitudinal studies were not linear, which is supported by the age‐group differences observed in the present study. At the distal site, the densitometric properties were best preserved in the younger well‐trained group, whereas at the mid‐tibia site, the bone properties were maintained or even improved in all athletes except those in the older less‐trained group. Furthermore, in accordance with Lauretani and colleagues,^(^ ^50^ ^)^ we found that the cross‐sectional linear trends derived from the baseline data were poor predictors of the longitudinal changes in bone (Supplemental Table S2). The differences in the results suggest that even in a relatively homogeneous group, predictions based on age alone poorly generalize the longitudinal processes that relate to modifications in individuals' bone characteristics.

The main limitation of this study is its observational nature, which only allows the reporting of associations, not causal relationships. We cannot totally exclude the possible influence on bone of other factors, such as genetics or diseases. It is noteworthy that many aging athletes continue to train and compete despite sustaining mild sports injuries and having potentially progressive diseases. Health‐related factors could, however, lead to an accelerated decline in bone strength, for example, by limiting the amount of systematic training. In the present sample, equal numbers of athletes in the well‐trained and less‐trained groups presented with prostate cancer, and their exclusion did not change the results. The athletes with cancer did not differ from the healthy athletes in their bone results or anthropometric or training characteristics. Moreover, review of the information on time of diagnosis and the treatment methods used also showed that the disease had no substantial effect on bone. Another potential limitation is that the group allocation into well‐trained and less‐trained athletes was based on self‐reported physical activity levels. However, with an athlete population accustomed to keeping exercise diaries on a regular basis, the probability of recall bias is likely to be lower than average. Furthermore, in the group allocation, special emphasis was placed on strength training, which was already low in the less‐trained group at baseline. However, as reported in the RCT,^(^ ^13^ ^)^ the previous strength training of the athletes had focused on strength endurance exercises (higher repetitions with low‐intensity loads) rather than the heavy and explosive exercises that were administered to all participants (experimental and control) along with the RCT. The original randomization grouping was not taken into account in the present analysis because it was not associated with 10‐year training status or the longitudinal changes in bone. Given the lengthy time frame and the independently performed training program that was fully provided for the control participants after completion of the trial, the RCT is unlikely to affect the current results. The less‐trained athletes were also highly active, and many were actively competing. We did, however, find that with specific intensive training, bone properties were better preserved, even in older participants. The present age group data are, however, exploratory, ie, hypothesis‐generating rather than hypothesis‐confirming. Given the considerable number of bone variables analyzed, the sample size was not sufficient for more fine‐grained age group analyses. To describe the mechanisms behind the bone changes, multiple outcomes of interest were preferred instead of a single outcome. Moreover, to avoid issues related to multiple testing, the results are presented both in raw form and corrected for multiple testing.

This study presents novel findings on the adaptability of the aging male skeleton to exercise and the extent to which regular intensive training counteracts age‐related changes in bone. The strengths of this study include the longitudinal design and the unique study population. As part of a larger research program including both cross‐sectional and experimental study designs, the present sample was carefully selected to represent high‐level competitive sprint athletes with years of habitual intensive training. Moreover, given the long‐term follow‐up, the retention rate was relatively high. A further strength of the study is the use of 3D imaging to detect changes in bone cross‐sectional geometry and volumetric density, although a higher‐resolution technology would have yielded even more detailed results. Furthermore, detailed mass distribution analysis has been reported in only a few earlier studies, and we are not aware of previous longitudinal data on masters athletes.

In conclusion, this longitudinal study suggests that regular strength and sprint training counteracts bone aging in middle‐aged and older men. Continued intensive training may hinder bone deterioration among even the oldest athletes, but more research is needed to confirm this. The present longitudinal findings further support the adaptability of aging bone to physical exercise and highlight the importance of a regular, intensive training stimulus for maintaining bone health. Further longitudinal studies should address the effects of combined strength and sprint/impact training on age‐related changes at the clinically important proximal femur site, also in female athletes and in sedentary aging people. Strength training and other high‐intensity training have become increasingly popular among older people, and exercises targeted at improving muscle force–generating capacity are highly recommended at all ages.

## Disclosures

All authors state that they have no conflicts of interest.

## Author Contributions

**Tuuli Suominen:** Formal analysis; investigation; methodology; visualization; writing‐original draft; writing‐review & editing. **Markku Alen:** Investigation; writing‐review & editing. **Timo Tormakangas:** Formal analysis; methodology; writing‐review & editing. **Hans Degens:** Writing‐review & editing. **Joern Rittweger:** Writing‐review & editing. **Ari Heinonen:** Writing‐review & editing. **Harri Suominen:** Conceptualization; funding acquisition; investigation; methodology; project administration; supervision; writing‐review & editing. **Marko Korhonen:** Conceptualization; funding acquisition; investigation; methodology; project administration; supervision; writing‐original draft; writing‐review & editing.

### Peer Review

The peer review history for this article is available at https://publons.com/publon/10.1002/jbm4.10513.

## Supporting information


**Appendix S1.** Supporting Information
