# Supplementary material for: Regular Strength and Sprint Training Counteracts Bone Aging: A 10‐Year Follow‐Up in Male Masters Athletes
Source: JBMR Plus. 2021 May 24;5(7):e10513. doi: 10.1002/jbm4.10513 (PMC8260815; doi:10.1002/jbm4.10513)
Supplement: Supplementary file 1 — Appendix S1. Supporting Information [file JBM4-5-e10513-s001.docx]

**SUPPLEMENTAL MATERIAL**


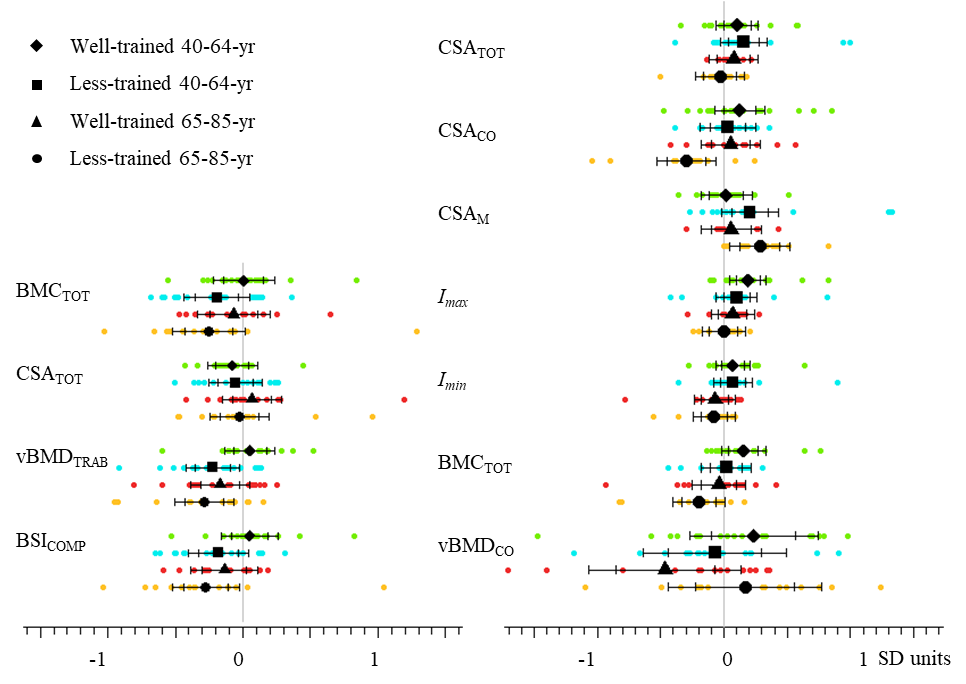


**Supplemental Fig. 1.** 10-year changes in distal tibia (*left panel*) and tibia midshaft (*right panel*) in well-trained and less-trained athletes by age group. Outcomes were standardized with respect to their baseline values. Individual data points, group means, and 95% CIs for unadjusted (narrower CIs) and M_eff_-Sidák multiple test-corrected (wider CIs) analyses are presented. Cases in the well-trained group with vBMD_CO_ = -3.36 (65-85-yr) and 2.54 (40-64-yr) were cropped from the figure on the right-hand side.

**
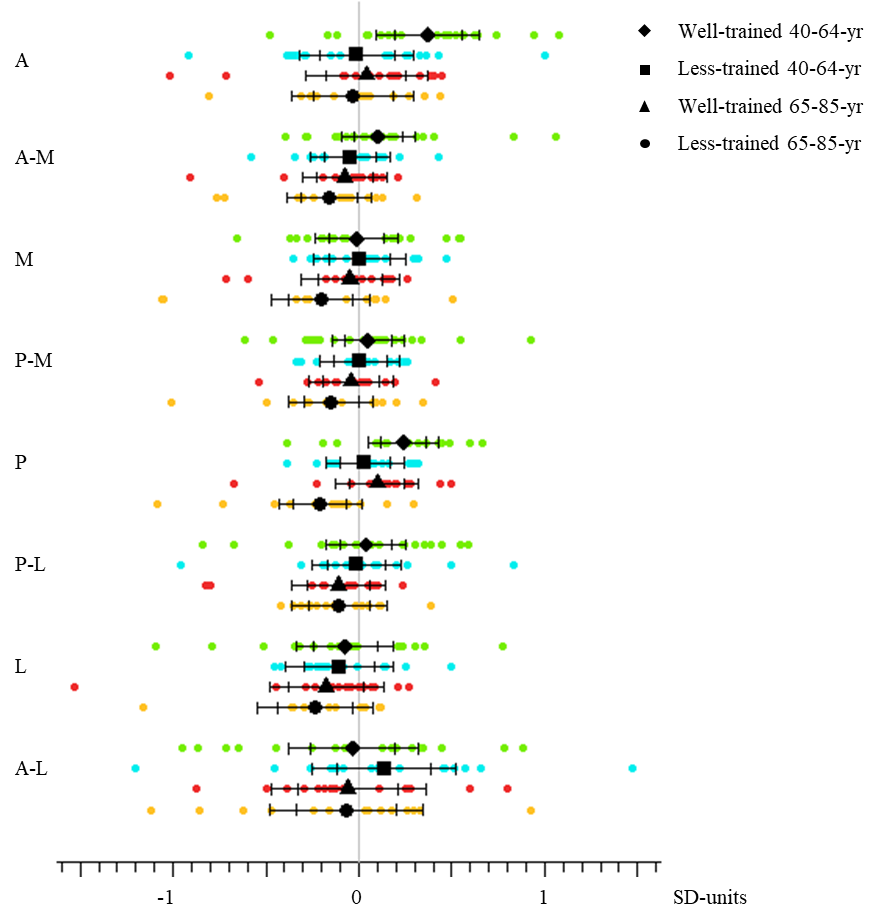
**

**Supplemental Fig. 2.** 10-year changes in polar mass distribution of the tibial shaft in well-trained and less-trained athletes by age group. Outcomes were standardized with respect to their baseline values. Individual data points, group means, and 95% CIs for unadjusted (narrower CIs) and M_eff_-Sidák multiple test-corrected (wider CIs) analyses are presented. (A = anterior, A-M = anteromedial, M = medial, P-M = posteromedial, P = posterior, P-L = posterolateral, L = lateral, A-L = anterolateral)

**Supplemental Table 1**. Baseline and follow-up physical, training and bone characteristics of well-trained and less-trained athletes by age group.

|  | **Baseline** | | | | | | **10 years** | | | |
| --- | --- | --- | --- | --- | --- | --- | --- | --- | --- | --- |
|  | 40-64 yr | | *p*-value | 65-85 yr | | *p*-value | 40-64 yr | | 65-85 yr | |
|  | WT (*n*=21) | LT (*n*=18) |  | WT (*n*=15) | LT(*n*=15) |  | WT(*n*=21) | LT(*n*=18) | WT(*n*=15) | LT (*n*=15) |
| Age, years | 54.3 (6.5) | 50.9 (7.8) |  | 69.8 (3.9) | 72.0 (5.6) |  | 64.2 (6.5) | 60.7 (7.8) | 79.6 (3.4) | 82.0 (5.5) |
| Height, cm | 177 (7) | 178 (7) |  | 171 (4) | 174 (5) |  | 176 (7) | 177 (7) | 170 (3) | 172 (5.2) |
| Mass, kg | 75.9 (6.0) | 74.7 (9.5) |  | 70.4 (7.2) | 71.9 (5.1) |  | 76.0 (7.8) | 77.2 (10.6) | 69.1 (6.4) | 71.3 (4.4) |
| LBM, kg | 65.6 (6.6) | 64.6 (6.5) |  | 59.8 (4.4) | 60.9 (4.1) |  | 64.2 (5.9)^a^ | 64.0 (8.1)^b^ | 58.4 (3.7)^b^ | 59.5 (4.4)^c^ |
| Muscle CSA, mm^2^ | 6901 (858) | 7338 (975) |  | 6428 (782) | 6225 (1315) |  | 7004 (958) | 7450 (931) | 6428 (782) | 6225 (1304) |
| 60-m sprint time, s | 8.00 (0.43)^a^ | 8.05 (0.51) |  | 8.86 (0.35) | 9.33 (0.85) |  | 8.74 (0.64)^d^ | 8.58 (0.68)^e^ | 10.4 (0.96)^f^ | 11.3 (2.9)^e^ |
| Training frequency, sessions/wk | 4.6 (1.3) | 4.2 (1.3) |  | 4.2 (1.1) | 4.5 (1.4) |  | 4.5 (1.5) | 3.2 (1.6) | 3.9 (0.7) | 3.4 (1.4) |
| Running and plyometrics, times/wk | 3.2 (1.1) | 2.7 (1.4) |  | 3.5 (1.9) | 3.3 (1.8) |  | 2.0 (0.6) | 0.9 (1.5) | 2.3 (0.6) | 0.6 (1.0) |
| Strength training, times/wk | 1.2 (0.5) | 0.5 (0.5) | .005 | 1.1 (0.8) | 0.8 (0.6) |  | 1.4 (0.8) | 0.9 (1.4) | 1.5 (0.5) | 0.5 (0.5) |
| **Tibia 5%** |  |  |  |  |  |  |  |  |  |  |
| BMC_TOT_ (mg/mm) | 450 (66) | 415 (83) | .043 | 395 (46) | 426 (52) |  | 450 (61) | 402 (83) | 391 (54) | 410 (62) |
| CSA_TOT_ (mm^2^) | 1204 (114) | 1181 (175) |  | 1182 (172) | 1254 (164) |  | 1192 (107) | 1173 (174) | 1193 (164) | 1251 (171) |
| vBMD_TRAB_ (mg/cm^3^) | 328 (32) | 302 (43) | .037 | 297 (42) | 298 (32) |  | 330 (30) | 294 (48) | 291 (44) | 287 (38) |
| BSI_COMP_ (g^2^/cm^4^) | 1.70 (0.39) | 1.48 (0.46) |  | 1.35 (0.30) | 1.47 (0.29) |  | 1.72 (0.37) | 1.41 (0.47) | 1.30 (0.33) | 1.36 (0.32) |
| **Tibia 50%** |  |  |  |  |  |  |  |  |  |  |
| CSA_TOT_ (mm^2^) | 600 (57) | 599 (89) |  | 582 (64) | 599 (48) |  | 607 (58) | 610 (88) | 587 (61) | 600 (54) |
| CSA_CO_ (mm^2^) | 431 (53) | 417 (55) |  | 395 (39) | 414 (33) |  | 436 (49) | 418 (61)^g^ | 398 (37) | 400 (36) |
| CSA_M_ (mm^2^) | 170 (29) | 183 (53) |  | 186 (56) | 184 (35) |  | 170 (32) | 191 (54)^g^ | 189 (63) | 200 (33) |
| *I_max_* (mg*cm) | 5154 (1086) | 5102 (1558) |  | 4592 (799) | 4906 (883) |  | 5373 (1084) | 5222 (1562)^g^ | 4671 (756) | 4903 (917) |
| *I_min_* (mg*cm) | 1818 (356) | 1837 (513) |  | 1734 (428) | 1863 (320) |  | 1846 (364) | 1889 (549)^g^ | 1706 (368) | 1832 (347) |
| BMC_TOT_ (mg/mm) | 526 (61) | 513 (71) |  | 484 (45) | 509 (38) |  | 535 (56) | 513 (76)^g^ | 481 (47) | 497 (40) |
| vBMD_CO_ (mg/cm^3^) | 1098 (24) | 1098 (30) |  | 1089 (24) | 1094 (21) |  | 1104 (15) | 1095 (36)^g^ | 1078 (47) | 1098 (22) |

Values are means (SD). ^a^ *n*=20, ^b^ *n*=11, ^c^ *n*=14, ^d^ *n*=16, ^e^ *n*=8, ^f^ *n*=9, ^g^ *n*=17. *p-*values for significant (*p*<0.005) between-group differences at baseline. WT= well-trained, LT= less-trained, LBM= lean body mass, Muscle CSA = muscle cross-sectional area, BMC_TOT_ = total BMC; CSA_TOT_ = total CSA; vBMD_TRAB_ = trabecular volumetric BMD; BSI_COMP_ = compressive bone strength index; CSA_CO_ = cortical CSA; CSA_M_ = subcortical/medullary CSA; *I_max_*, *I_min_* = density-weighted maximal and minimal moments of inertia; vBMD_CO_ = cortical vBMD.

**Supplemental Table 2.** Cross-sectional and longitudinal age trends in tibial bone traits.

| **Variable** | **Cross-sectional trend**  **(% per decade)^a^** | **Longitudinal trend**  **(% per decade)** |
| --- | --- | --- |
| **Distal tibia** *(n=69)* |  |  |
| BMC_TOT_ (mg/mm) | -3.5 (-6.9, -0.1) | -1.8 (-3.1, -0.6) |
| CSA_TOT_ (mm^2^) | 0.5 (-2.4, 3.3) | -0.4 (-1.2, 0.5) |
| vBMD_TRAB_ (mg/cm^3^) | -3.2 (-5.8, -0.5) | -1.8 (-2.7, -0.9) |
| BSI_COMP_ | -7.2 (-12.6, -1.9) | -3.1 (-5.1, -1.1) |
| **Tibial mid-shaft** *(n=68)* |  |  |
| CSA_CO_ (mm^2^) | **-3.4 (-5.9, -1.0)** | **-0.1 (-0.9, 0.8)** |
| *I_max_* (mg*cm) | **-6.5 (-11.4, -1.6)** | **2.3 (1.6, 3.5)** |
| *I_min_* (mg*cm) | -3.2 (-8.1, 1.7) | 0.2 (-0.9, 1.3) |
| BMC_TOT_ (mg/mm) | **-3.4 (-5.7, -1.0)** | **0.0 (-0.7, 0.7)** |
| vBMD_CO_ (mg/cm^3^) | -0.3 (-0.8, 0.2) | 0.0 (-0.5, 0.4) |

^a^Data from cross-sectional analysis at baseline. (mean, unadjusted 95% CI) Values in bold indicate a significant difference (*p*<0.05) between cross-sectional and longitudinal trends. Models are adjusted for age.
